# When a Head Is about to Burst: Attachment Mediates the Relationship Between Childhood Trauma and Migraine

**DOI:** 10.3390/ijerph17124579

**Published:** 2020-06-25

**Authors:** Natalia Kascakova, Jana Furstova, Jozef Hasto, Andrea Madarasova-Geckova, Peter Tavel

**Affiliations:** 1 Olomouc University Social Health Institute, Palacky University Olomouc, 771 11 Olomouc, Czech Republic; jana.furstova@oushi.upol.cz (J.F.); j.hasto.tn@gmail.com (J.H.); andrea.geckova@upjs.sk (A.M.-G.); peter.tavel@oushi.upol.cz (P.T.); 2 Psychiatric-Psychotherapeutic Outpatient Clinic, Pro Mente Sana, 811 08 Bratislava, Slovakia; 3 Department of Social Work, St. Elizabeth College of Health and Social Work, 811 02 Bratislava, Slovakia; 4 Faculty of Medicine, Department of Psychiatry, Slovak Medical University, 833 03 Bratislava, Slovakia; 5 Department of Health Psychology, Faculty of Medicine, Pavel Jozef Safarik University, 040 11 Kosice, Slovakia

**Keywords:** attachment, mediating effect, childhood trauma, migraine, health

## Abstract

Background: People exposed to childhood trauma show insecure attachment patterns and are more prone to chronic and pain-related conditions, including migraine. The aim of this study was to explore the mediating role of attachment in the association between childhood trauma and adulthood chronic health conditions, with a focus on migraine. Methods: Respondents from a representative sample of citizens of the Czech Republic (*n* = 1800, mean age: 46.6 years, 48.7% male) were asked to report various chronic and pain-related conditions, childhood trauma (The Childhood Trauma Questionnaire, CTQ), and attachment anxiety and avoidance (The Experience in Close Relationships Revised, ECR-R) in a cross-sectional, questionnaire-based survey conducted in 2016. Structural equation models (SEM) adjusted for sociodemographic variables were used to assess the relationship between childhood trauma, adulthood attachment, and adulthood chronic health conditions (migraine, other pain-related conditions, chronic health conditions other than pain, no chronic health complaints). Results: After adjusting for sociodemographic variables, SEM confirmed a significant mediation of the relationship between childhood trauma and migraine through adulthood attachment. There was no mediation effect of adulthood attachment found in other health complaints. Conclusion: This study highlights the mediation effect of attachment in the link between childhood trauma and migraine. Attachment-based therapeutic interventions can be useful in the treatment of patients with migraine.

## 1. Introduction

Folk proverbs often reflect some psychosomatic aspects of health complaints. The term “bursting” or “exploding” head is used by many people suffering from migraine. What could contribute to such feelings of “bursting” or “exploding”?

Based on attachment theory [1,2], many studies have tried to investigate how insecure attachment representations may influence health outcomes [3,4,5], including chronic pain [6,7] and migraine [8,9,10,11]. The link between childhood trauma and negative health outcomes [12,13,14,15,16], chronic pain [17,18], and migraine [19,20,21,22] is well established. On the other hand, not all individuals exposed to childhood trauma manifest somatic symptoms as adults. In this context, a protective effect of secure attachment has been discussed [23,24].

### 1.1. Health Complaints and Childhood Trauma

In the wake of the pioneering Adverse Childhood Experience (ACE) studies [12,13,14], many investigations summarized in meta-analytic studies [15,16] have confirmed the enduring impact of childhood trauma on poor adulthood health. From a meta-analytic study [17] and a recent German population study [18], the association between childhood trauma and chronic pain is known. Migraine, given its potential connection to childhood trauma, deserves special attention, because it is one of the most common causes of disability [25].

### 1.2. Insecure Attachment and Childhood Trauma

Attachment theory [1,2] provides a useful framework for understanding the relationship between early childhood experience and adult personality style of relating to others. According to attachment theory, infants develop expectations about caregivers’ availability and responsiveness based on the quality of parental care they receive. Attachment researchers have identified four attachment styles that vary along two dimensions: attachment anxiety (worry over the availability, responsiveness, and positive regard of others) and attachment avoidance (discomfort with closeness and interdependence) [26]. People with “secure” attachment are low in both dimensions, they have a sense of worthiness and an expectation that other people are generally accepting and responsive. The following three attachment styles are considered as insecure: preoccupied, dismissive, and fearful. “Preoccupied” persons are high in anxiety but low in avoidance; they feel themselves as unworthy and are hypervigilant to threats. “Dismissive” persons are high in the avoidance dimension but low in anxiety; for them, the independency is the most important, and when threatened they use deactivating strategies and rely on themselves. “Fearful” persons, high in anxiety and avoidance, experience themselves as unworthy and tend to have high expectations from others, but concurrently they consider others to be unpredictable and rejecting [27].

Experience of early abuse and neglect can affect a child’s internal working models and subsequently the child’s relationship to others by seeing them as mistrustful, unpredictable, and not loving. Several studies suggest that maltreated children show insecure attachment patterns [28,29]. Attachment insecurity in university students was associated with childhood trauma [30,31] and was linked to experiencing depression and using destructive behavior [31]. A meta-analytic study [32], a prospective study [33], and a study comprising population and clinical samples [34] brought clear evidence that adults with a history of abuse and neglect have mostly insecure states of mind, in the sense of preoccupied, dismissive, and fearful attachment.

### 1.3. Insecure Attachment and Health

There is a lot of evidence suggesting that insecure attachment representations are linked to dysregulated physiological responses to stress, risky health behaviors, susceptibility to physical illness, and poorer disease outcomes [3,4,5,35,36].

Insecure attachment seems to be a risk factor for some chronic pain conditions [7,37] and for poorer management of these conditions [38]. In a population-based study [39], subjects with chronic pain were more likely to report an insecure, mostly preoccupied, attachment style. Experiencing pain represents a stressor that activates the attachment system, and people with an insecure attachment style can respond to it in a nonadaptive way [40]. Anxiously attached (preoccupied) individuals tend to catastrophize their pain and emphasize their negative feelings to elicit more support from others, while avoidantly attached (dismissing) individuals are likely to ignore pain signals or to appraise them as posing little threat [6,7]. Anxiously-avoidantly attached (fearful) individuals are characterized by alternation of hyperactivating and deactivating strategies in coping with pain [40].

### 1.4. Attachment as a Mediator

A mediating effect of insecure attachment between childhood trauma and somatization was stated in a population study of couples [41]. However, the effect was found only in females. Another study, a computer-based survey with 691 participants, showed a mediation effect of insecure attachment between childhood trauma and somatic self-reported symptoms, including headache [42].

**Figure 1:**
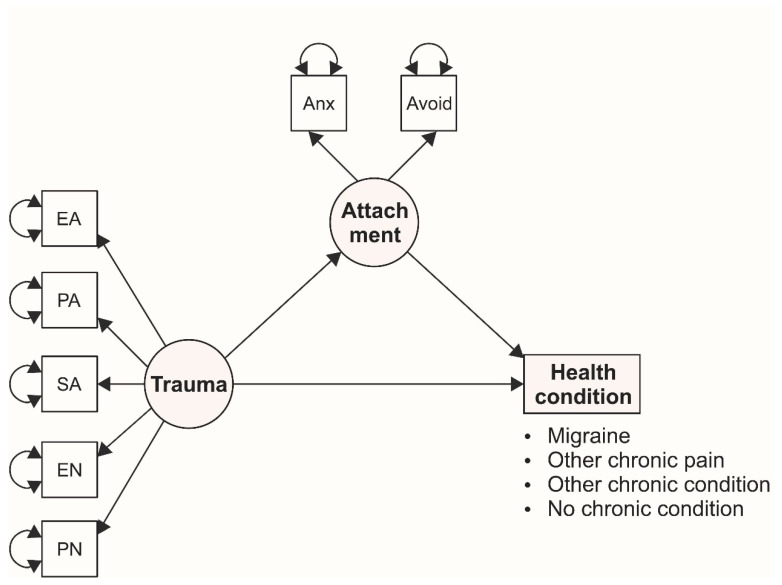
Hypothesized mediation models for various adulthood health conditions. Note: Trauma = latent variable assessing childhood trauma, Attachment = latent variable assessing adulthood attachment; EA = emotional abuse, PA = physical abuse, SA = sexual abuse, EN = emotional neglect, PN = physical neglect; Anx = anxiety, Avoid = avoidance.

Thus, the aim of this study was to explore the potential mediating effect of adulthood attachment in the link between childhood trauma and various adulthood health conditions (migraine, other pain-related conditions, chronic health conditions other than pain, no chronic health complaints). To assess this effect, we used data from a representative sample of the Czech Republic. Our hypothesis was that the effect of childhood trauma on the studied chronic health conditions is mediated through insecure attachment (Figure 1).

## 2. Methods

### 2.1. Sample

A pilot study on 206 respondents was performed prior the study with aim of checking the readability of the questionnaire. Then, 2184 randomly selected respondents from a list of inhabitants of the Czech Republic, stratified by gender, age, and 14 regions, were contacted by trained administrators and asked to participate in a health survey. No compensation was offered. Of those asked to participate, 384 refused, more men and younger age groups, mostly due to a lack of time, nonconfidence, the length of the questionnaire, or reluctance. Finally, data from 1800 respondents were collected by the administrators using face-to-face interviews in the respondents’ households during September and November 2016. The selected group of 1800 participants is a representative sample of the population of the Czech Republic over the age of 15 in relation to sex (48.7% of men), age composition (age 15 to 90 years old, mean age: 46.61), and regional affiliation. 

No data was missing in the representative sample. Respondents agreed to participate in the study by signing an informed consent prior to the study. This study was approved on 14 June 2016 by the Ethical Scientific Committee of Palacky University Olomouc (No 2016/3) and conducted in accordance with the protection of personal data law (Act. No 101/2000 Coll.).

**Figure 2:**
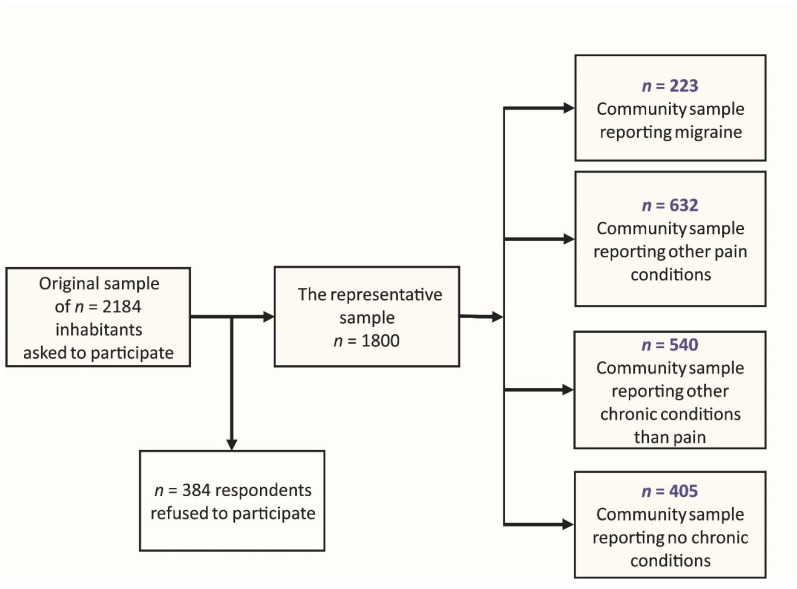
Scheme describing the final sample selection and research groups.

For the purpose of this study, four research groups were created (Figure 2): (1) Respondents reporting migraine. The respondents could also concurrently suffer from some other pain or chronic conditions (*n* = 223); (2) Respondents reporting some other pain-related condition excluding migraine (back pain, arthritis, pelvic pain, pain of unclear origin). These respondents could concurrently suffer from some other chronic—not only pain-related—conditions (*n* = 632); (3) Respondents reporting some chronic conditions other than pain (e.g., hypertension, asthma, diabetes, allergy, etc.) (*n* = 540); (4) Respondents reporting no chronic conditions, that is, “healthy” respondents (*n* = 405).

### 2.2. Measures

#### 2.2.1. Sociodemographic Data

Participants reported gender (male or female), age (continuous, categorized for analyses purposes), marital status (single, married, divorced, widowed, or unmarried partner), education (primary, skilled operative, high school graduate, and college), and economical status (student, disabled, employed, entrepreneur, in household, unemployed, and pensioner).

#### 2.2.2. Long-Term Health Complaints 

Long-term health complaints were measured by the item “Do you have any long-lasting disorder or disability? Please, mark all possibilities which are related to you”. Respondents chose from the following list: ischemic heart disease, hypertension, cerebral insult/hemorrhage, chronic pulmonary disease, asthma, cancer, diabetes, obesity, arthritis, back pain, gastric and duodenal ulcer, inflammatory bowel disease, dermatitis (eczema), allergy, migraine, pain of unclear origin, pelvic pain—in women, diseases of thyroid gland, anxiety, other, or no disease.

#### 2.2.3. Childhood Trauma

The Childhood Trauma Questionnaire (CTQ) is a retrospective self-report measuring the severity of five different types of childhood trauma: emotional abuse (EA), physical abuse (PA), sexual abuse (SA), emotional neglect (EN), and physical neglect (PN) [43]. Each subscale has five items rated on a five-point Likert-type scale with response options ranging from (1) never true to (5) very often true. The Czech version of the CTQ has been shown to be both reliable and valid. The Cronbach’s alpha for the whole questionnaire was 0.92 and for the individual subscales varied from 0.64 to 0.92 [44]. We used Walker’s procedure of severity ratings in the present study [45]. According to Walker’s approach, PA and PN include all cases from “slight to moderate” up to “extreme” childhood trauma (cut-off score 8), and SA and EN include all cases from “moderate to severe” up to “extreme” childhood trauma (8 for SA, 15 for EN). For EA, the cut-off point is in the middle of the “slight to moderate” level (cut-off score 9).

Analysis of the factor structure of the CTQ as a latent variable for childhood trauma revealed that the five subscales—EA, PA, SA, EN, and PN—used for measuring childhood maltreatment as a single factor solution showed significant loadings above 0.65 (*p* < 0.001).

#### 2.2.4. Attachment Anxiety and Avoidance

The Experiences in Close Relationships Revised (ECR-R) questionnaire is a 36-item, self-report measure of adult attachment represented by attachment anxiety and attachment avoidance. It uses a Likert scale from 1 (Strongly agree) to 7 (Strongly disagree) [46]. In a meta-analysis of five self-report attachment measures, the ECR-R was the measure with the highest average reliability, which was relatively unaffected by characteristics of sample and setting [47]. In this study, the short Czech version ECR-R-16 was used with a Cronbach’s alpha of 0.87 for the anxiety subscale and 0.91 for the avoidance subscale [48,49].

### 2.3. Statistical Analyses

All the statistical analyses were performed using the R software, version 3.6.3 (R Foundation for Statistical Computing, Vienna, Austria) [50]. Frequencies, percentages, means, and standard deviations (SD) were used to describe the sociodemographic characteristics, prevalence of reporting various health conditions, and prevalence of childhood trauma. Unadjusted logistic regression was employed to model the odds of various adulthood health conditions, depending on sociodemographic variables. To assess the correlation between childhood trauma and adulthood attachment, Spearman correlation coefficients were evaluated. Structural equation models (SEM) were used to investigate the mediating relationship between childhood trauma, adulthood attachment, and reported adulthood health conditions. Childhood trauma and adulthood attachment were modeled as latent variables measured by the subscales of the Childhood Trauma Questionnaire (CTQ) and the Experiences in Close Relationships Revised (ECR-R) scale, respectively. The analysis was conducted in two steps: (1) testing the direct effect of childhood trauma on the reported adulthood health conditions, and (2) testing the mediating effect of adulthood attachment on the relationship between childhood trauma and the reported adulthood health conditions. A separate SEM model was fitted for each of the adulthood health conditions. To measure the effect of childhood trauma and adulthood attachment style, latent variables were used. All the SEM models (i.e., regression formulas in the models) were adjusted for sociodemographic variables. For fitting the SEM models, the R Lavaan package [51] was used. Parameters were estimated using the diagonally weighted least squares (DWLS) method, based on a polychoric correlation matrix. The models were evaluated based on the following fit indices: a comparative fit index (CFI) > 0.95, Tucker–Lewis index (TLI) > 0.95, root mean square error of approximation (RMSEA) < 0.06, and standardized root mean square residual (SRMR) < 0.08 were considered a good fit [52]. The mediation effect was tested in the Lavaan package with bootstrap standard errors. Significance level was set at the level of *p* < 0.05 for all statistical significance testing.

## 3. Results

### 3.1. Sociodemographic Characteristics

The sociodemographic characteristics of the sample are shown in Table 1. Participants reported suffering from migraine (12.4%), other chronic pain conditions (35.1%), chronic conditions other than pain (30.0%), or reported no chronic conditions (22.5%). There were 129 respondents who reported migraine together with at least one other type of pain (57.8% of those who reported migraine). Unadjusted logistic regression showed that gender was a significant predictor of migraine and no illness, with males having lower odds of reporting migraine and higher odds of reporting no illness (Table 1). The only other significant predictor of reporting migraine was economic status; respondents in household (including maternity leave) presented higher odds of suffering from migraine than students. All the studied sociodemographic characteristics except for gender showed to be significant predictors of other types of chronic pain. In contrast, no sociodemographic characteristics, except economic activity, affected the odds of suffering from other chronic illnesses. The odds of reporting no chronic illness were affected by all the sociodemographic characteristics presented in Table 1.

Descriptive characteristics of childhood trauma and adulthood attachment are presented in Table 2. The prevalence of individual types of childhood trauma was measured according to Walker’s clinical cut-off scores [45].

The correlation between childhood trauma and adulthood attachment was found to be low to moderate, with values from 0.06 to 0.37. In all the research groups, attachment anxiety had higher correlations with childhood emotional abuse, while attachment avoidance was more strongly correlated with emotional and physical neglect. The group reporting migraine is the only one in which correlations between emotional and physical neglect and attachment anxiety and avoidance are approximately the same (approximately 0.30). The correlation coefficients evaluated within the research groups are presented in Table 3.

### 3.2. Childhood Trauma Predicts Adulthood Health 

The effect of childhood trauma on adulthood health was assessed using SEM models and adjusting for all the sociodemographic variables presented in Table 1, that is, gender, age, marital status, education, and economic status. All the studied SEM models showed acceptable values of CFI and TLI indices and of RMSEA and SRMR (see Table 4). The latent factor, childhood trauma, had a significant direct effect on reporting migraine and reporting no chronic conditions (Table 4). Higher exposure to childhood maltreatment increased the likelihood of reporting migraine and decreased the likelihood of reporting no chronic conditions. 

### 3.3. The Effect of Childhood Trauma on Migraine is Mediated by Adulthood Attachment

**Figure 3:**
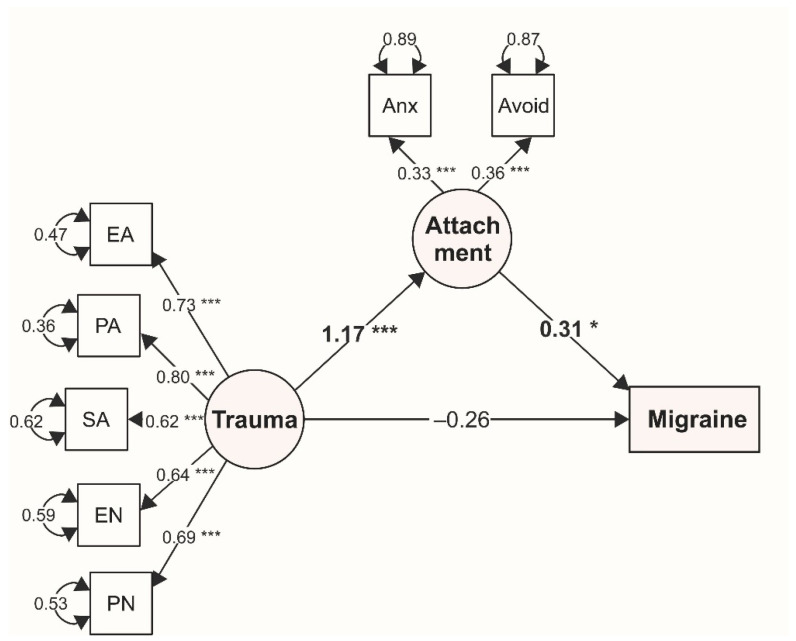
Mediation model between childhood trauma, adulthood attachment, and migraine, adjusted for sociodemographic variables. The mediation path coefficients are highlighted in boldface. Note: Trauma = latent variable assessing childhood trauma, Attachment = latent variable assessing adulthood attachment; EA = emotional abuse, PA = physical abuse, SA = sexual abuse, EN = emotional neglect, PN = physical neglect; Anx = anxiety, Avoid = avoidance. *** *p* < 0.001, * *p* < 0.05.

The indirect effect of childhood trauma on adulthood health, mediated by adulthood attachment, was assessed in SEM models adjusted for all the sociodemographic variables presented in Table 1, that is, gender, age, marital status, education, and economic status. The models showed an acceptable fit to the data, with acceptable values of CFI, TLI, RMSEA, and SRMR (see Table 5). In the final SEM model (Figure 3), adulthood attachment fully mediates the effect of childhood trauma on reporting migraine (bootstrap *p*-value for the total effect *p* = 0.009, for the total indirect effect *p* = 0.041). Higher exposure to childhood maltreatment increased the likelihood of reporting insecure attachment in adulthood; furthermore, the insecure attachment style resulted in a higher likelihood of reporting migraine. The direct effect of childhood trauma on reporting migraine lost its significance. The mediation effect of adulthood attachment was not found in other health conditions. Childhood trauma had a significant direct effect on adulthood attachment in all the assessed SEM models; however, there was no relationship found between attachment and health conditions other than migraine (Table 5).

## 4. Discussion

This study explored the mediating role of attachment on the relationship between childhood trauma and adulthood health condition. After adjusting for sociodemographic variables, SEM confirmed significant mediation of the relationship between childhood trauma and migraine through adulthood attachment. There was no mediation effect of adulthood attachment found in other health complaints.

The mediation models were assessed separately for people reporting migraine, other pain-related conditions, other chronic conditions, and people reporting no chronic conditions. The first step of our analyses was to assess the direct effect of childhood trauma on health. There is a lot of evidence from population studies about the association between childhood trauma and poor health [12,13,14,15,53], including chronic pain-related conditions [17,18] and migraine [19,20,21,22,54,55,56,57]. The first step of SEM analysis in our study confirmed the direct effect of reporting childhood trauma on reporting migraine, which is in line with findings that more than 50% of individuals with migraine report some type of childhood trauma [19] and that people reporting childhood trauma have higher odds of suffering from migraine compared with people without a history of maltreatment [22,54]. However, in our study, no direct effect was found between childhood trauma and adulthood pain conditions other than migraine and other chronic conditions. This is in contrast with large ACE studies [12,13,14,53] and meta-analytic studies [15,18] confirming links between childhood trauma and various chronic conditions, or between childhood trauma and chronic pain [17,18,58]. In a recent study analyzing the same Czech representative sample, a group of chronic pain conditions (including migraine) with/without anxiety was assessed, and the link between chronic pain and childhood trauma was confirmed [58]. The negative relationship between childhood trauma and no chronic conditions in our study revealed the empirical knowledge and experience that people raised in good conditions early in life, without excessive stress caused by abuse and neglect, are more likely to have good health in later life. A recent investigation of Cicchetti et al. [59] showed significant differences between maltreated and nonmaltreated children in methylation across the epigenome, which is associated with increased risk for adverse physical and mental health outcomes in maltreated children.

As a second step, we tested the mediating effect of adulthood attachment on the relationship between childhood trauma and the reported adulthood health conditions. The mediation model showed that adulthood attachment fully mediated the effect of childhood trauma on migraine, in contrast to other chronic conditions. Why is attachment important as a mediator in the relationship between childhood trauma and migraine? Could emotional distress related to attachment insecurity be this “bursting” or “exploding” head element in migraine?

Attachment insecurity in individuals with migraine was found in studies with child or adolescent patients [9,10,11,60] and in adult patients [8,61,62] with higher rates of insecure-ambivalent and insecure-avoidant attachment and lower rates of secure attachment. The study of Waldinger et al. [41] supported the hypothesis that insecure attachment mediates the link between childhood trauma and somatization, including headache, in females, whereas in males both childhood trauma and attachment had an indirect effect on somatization. Similarly, the study of Lin et al. [42] showed that differences in attachment anxiety shape the association between adverse childhood experiences and adult somatic symptoms, including headache.

Individuals with high attachment anxiety, due to their feelings of unworthiness and excessive need of reassurance in relationships, are prone to experiencing many interpersonal interactions as a source of distress [40]. Being daily under stress may be a factor in the onset of migraine; it can act as a trigger for individual migraine attacks [63,64], and it may play a role in the progression of migraine to a chronic migraine syndrome [63].

The psychoanalytic view on psychogenesis of migraine was illustrated by Fromm-Reichmann [65] in an original article from 1937: “Experience with eight cases of migraine (two men and six women) has given me the impression that they all were patients suffering from unresolved ambivalence; they could not stand to be aware of their hostility against beloved persons; therefore they unconsciously tried to keep this hostility repressed, and finally expressed it by the physical symptoms of migraine“. This psychoanalytic interpretation that patients with migraine have strong hostile impulses toward highly intellectual persons resulted in guilty feelings turned against themselves [65] was further elaborated by Franz Alexander in his theory of psychosomatic medicine [66]. The theory has been supported by a recent finding of inhibited anger in children with severe migraine [67] and a greater level of suppressed anger and hostility in subjects suffering from headache [68]. Perozzo et al. [69] supposed that the lack of anger control in patients with headache is related more to an increase in the experience of anger as feelings of resentment, mistrust, or frustration in relationships rather than representing a direct expression of anger toward other people or objects. Negative affectivity is connected to alexithymia [70], a concept that is also studied in its association to migraine [71,72]. Natalucci et al. [71] suggest that the association between alexithymia and headache could be moderated by insecure attachment or by incomplete development of emotive competency, which may result in a deficit in emotional regulation and expression. Although we cannot apply theories of suppressed or repressed negative feelings for all patients with migraine, from the clinical therapeutic treatment of patients with migraine, we can often follow that relational conflicts (situated in reality or in an internal world) connected to some unpleasant emotional state may act as a trigger of a migraine attack.

According to bio-psycho-social concept of illness [73,74], a new integrative four-cluster model of mind–body interrelationships was proposed [75]. In this model, migraine is in a cluster of stress-exacerbated diseases, where chronic stress and emotional processes play a substantial role in the exacerbations [75]. It seems that the impact of stress and medication overuse and other risk factors can lead to a reduced threshold for an induction of headache and abet the transformation of episodic migraine into chronic daily headache [76]. The mechanism of central sensitization—the amplification of neural signaling in the central nervous system contributing to hyperalgesia [77]—plays an important role in chronic migraine and other chronic pain-related conditions, which are often in comorbidity with migraine [78], as was also found in our study, where more than half of respondents reported at least one other pain condition. The stress-induced hyperalgesia underlying many chronic pain conditions, including migraine, is highly associated with childhood trauma and with attachment insecurity [79,80]. Attachment-related neurobiological research suggests an attenuated regulatory functioning of the right orbitofrontal cortex in people with attachment insecurity [81]. Orbitofrontal dysfunction is present in patients with chronic migraine and medication overuse [82]. Reduced inhibitory functioning of the prefrontal cortex is a possible cause for disinhibition of the pain-related sensory cortices in migraine [83].

Later life stress represents another negative etiopathogenetic factor. There is well-established empirical evidence that exposure to traumatic events increases central sensitization in patients with chronic pain [84]. We did not focus on later life traumatic events or on posttraumatic stress disorder (PTSD) in this study, but we can hypothesize that some of our respondents reporting childhood trauma and migraine could be revictimized in adulthood, especially those with a history of childhood trauma and with high attachment anxiety [85]. According to the conclusions of a study exploring PTSD in migraine [86], PTSD is more common in chronic than in episodic migraine. A cross-sectional study with individuals with migraine [87] showed that PTSD was a robust predictor of migraine, whereas trauma exposure alone was not. Another study showed that childhood trauma, life events, and alexithymia were associated with chronic migraine and, moreover, with medication overuse [88].

The effect of childhood trauma on attachment insecurity found in all the research groups in our study supports the extensive findings in this field in samples of children [28,29], university students [30,31], and the adult population as well as in clinical samples [32,33,34]. The effect of chronic stress and fear following child abuse and neglect on the hypothalamic–pituitary–adrenal axis dysfunction [88] makes insecurely attached individuals more susceptible to stress and more prone to risky health behaviors, physical and mental illnesses, and poorer disease outcomes [3,4,5,35,36]. This has also been proved in patients with migraine, where an insecure style of attachment was found to be a significant predictor of higher levels of migraine-related disability [62]. Another study showed that insecure attachment may exacerbate anxiety in children and adolescents with migraine [11]. Anxiety in relation to pain potentiates higher sensitivity to pain and can contribute to pain chronification [89,90]. 

We can assume that there is an association between childhood trauma, attachment insecurity, and migraine, regardless of the extent to which they are involved in the etiopathogenesis and the course and treatment outcomes of migraine. Patients with frequent and chronic migraine in particular should be screened for the occurrence of childhood trauma and attachment insecurity. In patients with chronic pain and a history of early stress, multimodal therapeutic approaches comprised of, for example, education about mechanisms maintaining chronic pain, better self-awareness training, fostering positive self-body image, relaxation techniques training, and so forth, can be useful [91]. Patients with a higher level of attachment insecurity (especially those with a high level of both anxiety and avoidance) can be very persistent and insistent in their pain complaints. The integration of an attachment-based approach in the treatment of patients with attachment insecurity can help to better understand their problematics and manage treatment [92,93]. A psychodynamic approach, with identifying of conflicts in close relationships and negative emotional states which contribute to the daily distress in people with migraine, has been shown to be effective in improving the disease parameters [94].

### Strengths and Limitations

The strength of this study is that it is based on a representative sample. A community sample offers the advantage of examining the link between childhood trauma, attachment, and health complaints in the whole population. This approach might bring a better overview of the situation than studies based only on patient data from medical facilities. 

Migraine status was based on self-report of a diagnosis, and this could be confused, for example, with tension headache, which may be less strongly associated with some childhood adversities, as was found in a study comparing migraine with tension headache [55]. Related to the above-mentioned limitation is that we were unable to determine the age of onset, frequency, severity, or type of migraine. On the other hand, the results of national studies suggest that the assessment of chronic conditions by self-reports is a valid option in mental–physical comorbidity research [95].

Another limitation could be confounding factors. The results were controlled for several sociodemographic factors, but they were not controlled for the presence of other pain-related conditions, the occurrence of life stressors in later life, or PTSD symptomatology in respondents reporting migraine. This could have affected the results. 

## 5. Conclusions

This study showed that the direct effect of childhood trauma on migraine is outweighed by the mediation effect of adulthood attachment. This effect was not found for other pain-related conditions or other chronic health complaints. Thus, we can assume that there is an association between childhood trauma, attachment insecurity, and migraine, regardless of the extent to which they are involved in the etiopathogenesis, course, and treatment outcomes of migraine. Integration of an attachment-based approach to the treatment of migraine patients with attachment insecurity can help to better understand their problematics and manage the treatment.

## Figures and Tables

**Table 1:** Sociodemographic characteristics of the sample and unadjusted odds ratios acquired from logistic regression.

Health Condition		Migraine	Other Pain	Other Illness	No Illness
	***n*** **(%)**	223 (12.4%)	632 (35.1%)	540 (30.0%)	405 (22.5%)
Sociodemographic Group		**OR (95% CI)**	**OR (95% CI)**	**OR (95% CI)**	**OR (95% CI)**
Total	1800 (100.0)				
Gender					
1. Female	923 (51.3)	Ref	Ref	Ref	Ref
2. Male	877 (48.7)	**0.39 (0.29, 0.53)**	0.90 (0.74, 1.09)	1.20 (0.98, 1.46)	**1.62 (1.30, 2.03)**
Age					
Mean (± SD ^1^)	46.4 (± 17.4)	0.99 (0.98, 1.00)	**1.04 (1.03, 1.05)**	1.00 (0.99, 1.01)	**0.95 (0.95, 0.96)**
Marital status					
1. Single	439 (24.4)	Ref	Ref	Ref	Ref
2. Married	929 (51.6)	0.89 (0.63, 1.26)	**2.99 (2.28, 3.93)**	1.01 (0.79, 1.29)	**0.33 (0.25, 0.42)**
3. Divorced	158 (8.8)	1.08 (0.64, 1.84)	**3.16 (2.13, 4.69)**	0.85 (0.57, 1.28)	**0.33 (0.21, 0.52)**
4. Widow/Widower	133 (7.4)	0.92 (0.51, 1.66)	**3.98 (2.63, 6.03)**	0.81 (0.53, 1.26)	**0.26 (0.15, 0.44)**
5. Unmarried mate	141 (7.8)	1.04 (0.60, 1.82)	1.47 (0.94, 2.30)	1.00 (0.66, 1.51)	0.73 (0.49, 1.10)
Education level					
1. Primary	141 (7.8)	Ref	Ref	Ref	Ref
2. Skilled operative	442 (24.6)	1.03 (0.59, 1.79)	1.01 (0.69, 1.48)	0.74 (0.49, 1.12)	1.53 (0.88, 2.66)
3. High school, graduated	854 (47.4)	0.80 (0.47, 1.36)	**0.61 (0.43, 0.88)**	1.08 (0.74, 1.59)	**2.26 (1.34, 3.79)**
4. College/University	363 (20.2)	0.98 (0.55, 1.73)	**0.55 (0.37, 0.82)**	1.04 (0.69, 1.59)	**2.39 (1.38, 4.13)**
Economic status					
1. Student	178 (9.9)	Ref	Ref	Ref	Ref
2. Disabled	63 (3.5)	1.15 (0.48, 2.76)	**9.46 (4.84, 18.47)**	0.64 (0.34, 1.21)	**0.05 (0.01, 0.21)**
3. Employed	939 (52.2)	1.28 (0.78, 2.11)	**2.89 (1.81, 4.62)**	0.77 (0.55, 1.08)	**0.54 (0.39, 0.76)**
4. Entrepreneur	170 (9.4)	0.94 (0.48, 1.84)	**3.67 (2.12, 6.35)**	**0.63 (0.40, 0.99)**	**0.61 (0.39, 0.96)**
5. In household	38 (2.1)	**2.45 (1.02, 5.92)**	**2.89 (1.26, 6.63)**	**0.39 (0.16, 0.94)**	0.61 (0.29, 1.32)
6. Unemployed	45 (2.5)	1.46 (0.57, 3.69)	**5.18 (2.47, 10.87)**	**0.44 (0.20, 0.96)**	**0.43 (0.20, 0.93)**
7. Pensioner	367 (20.4)	0.70 (0.39, 1.28)	**9.92 (6.06, 16.23)**	0.74 (0.50, 1.07)	**0.06 (0.03, 0.11)**

^1^ SD = standard deviation; OR = odds ratio; CI = confidence interval; Ref = reference category; boldface values are significant (*p* < 0.05).

**Table 2:** Prevalence of individual types of childhood trauma, and descriptive characteristics of childhood trauma and adulthood attachment in the research groups.

Scale	Migraine		Other Pain		Other Illness		No Illness	
	*n* (%) ^†^	Mean (± SD)	*n* (%) ^†^	Mean (± SD)	*n* (%) ^†^	Mean (± SD)	*n* (%) ^†^	Mean (± SD)
Childhood trauma (CTQ)								
Emotional abuse	46 (20.6)	7.6 (3.3)	103 (16.3)	7.2 (3.0)	76 (14.1)	6.9 (2.7)	37 (9.1)	6.5 (2.2)
Physical abuse	31 (13.9)	6.0 (2.3)	82 (13.0)	6.0 (2.3)	60 (11.1)	5.8 (2.1)	37 (9.1)	5.6 (1.9)
Sexual abuse	21 (9.4)	5.6 (1.9)	41 (6.5)	5.5 (1.8)	39 (7.2)	5.6 (2.2)	25 (6.2)	5.4 (1.6)
Emotional neglect	57 (25.6)	11.4 (5.1)	128 (20.3)	10.6 (4.5)	85 (15.7)	10.0 (4.4)	67 (16.5)	9.9 (4.5)
Physical neglect	85 (38.1)	7.4 (2.9)	254 (40.2)	7.6 (2.8)	188 (34.8)	7.3 (2.6)	115 (28.4)	6.9 (2.6)
Adulthood attachment (ECR-R)								
Anxiety		21.9 (10.5)		18.8 (9.3)		19.5 (9.8)		18.4 (9.5)
Avoidance		24.6 (11.8)		24.2 (12.2)		23.4 (11.5)		23.2 (12.5)

^†^ The individual types of childhood trauma dichotomized according to Walker’s clinical cut-off scoring [45]. Means and standard deviations (SD) computed from the gross scores of the subscales. CTQ: Childhood Trauma Questionnaire; ECR-R: Experiences in Close Relationships Revised.

**Table 3:** Spearman correlation coefficients between individual types of childhood trauma and adulthood attachment in the research groups.

Adulthood Attachment (ECR-R)	Migraine		Other Pain		Other Illness		No Illness	
	Anxiety	Avoidance	Anxiety	Avoidance	Anxiety	Avoidance	Anxiety	Avoidance
**Childhood trauma (CTQ)**								
Emotional abuse	0.20 **	0.11	0.26 ***	0.11 **	0.23 ***	0.18 ***	0.29 ***	0.20 ***
Physical abuse	0.15 *	0.09	0.14 ***	0.10 *	0.09 *	0.19 ***	0.20 ***	0.17 ***
Sexual abuse	0.15 *	0.21 **	0.21 ***	0.20 ***	0.06	0.16 ***	0.17 ***	0.24 ***
Emotional neglect	0.32 ***	0.34 ***	0.24 ***	0.32 ***	0.21 ***	0.37 ***	0.19 ***	0.35 ***
Physical neglect	0.33 ***	0.29 ***	0.14 ***	0.32 ***	0.22 ***	0.35 ***	0.13 *	0.35 ***

* *p* < 0.05, ** *p* < 0.01, *** *p* < 0.001.

**Table 4:** Structural equation models (SEM) analyzing the direct effect of childhood trauma (CTQ) on reported adulthood health conditions.

Path	Standardized Parameter Estimate	Standard Error	*p*-Value
Childhood trauma → Migraine	**0.101**	**0.039**	**0.010**
Childhood trauma → Other pain	0.050	0.032	0.113
Childhood trauma → Other chronic condition	−0.013	0.034	0.697
Childhood trauma → No chronic condition	**−0.128**	**0.037**	**0.001**
Model fit indices			
CFI	0.969–0.970		
TLI	0.986–0.987		
RMSEA (90% CI)	0.057 (0.050, 0.064) ^†^		
SRMR	0.062–0.065		

Models adjusted for gender, age, marital status, education and economic status. ^†^ Pooled interval of 90% CIs across all models. CFI: comparative fit index; TLI: Tucker–Lewis index; RMSEA: root mean square error of approximation; SRMR: standardized root mean square residual; boldface values are significant (*p* < 0.05).

**Table 5:** Structural equation models (SEM) analyzing the indirect effect of childhood trauma (CTQ) on reported adulthood health, mediated by adulthood attachment (ECR-R).

Path	Standardized Parameter Estimate	Standard Error	*p*-Value
Health condition: migraine			
Childhood trauma → Attachment	**1.166**	**0.219**	**<0.001**
Attachment → Migraine	**0.312**	**0.124**	**0.012**
Childhood trauma → Migraine	−0.262	0.182	0.151
Health condition: other pain			
Childhood trauma → Attachment	**1.162**	**0.218**	**<0.001**
Attachment → Other pain	−0.127	0.099	0.202
Childhood trauma → Other pain	0.197	0.128	0.122
Health condition: other chronic condition			
Childhood trauma → Attachment	**1.162**	**0.218**	**<0.001**
Attachment → Other chronic condition	−0.002	0.099	0.986
Childhood trauma → Other chronic condition	−0.012	0.122	0.922
Health condition: no chronic condition			
Childhood trauma → Attachment	**1.163**	**0.218**	**<0.001**
Attachment → No chronic condition	−0.074	0.102	0.469
Childhood trauma → No chronic condition	−0.041	0.130	0.754
Model fit indices			
CFI	0.954–0.955		
TLI	0.976		
RMSEA (90% CI)	0.059 (0.053, 0.065) ^†^		
SRMR	0.061–0.064		

Models adjusted for gender, age, marital status, education and economic status. ^†^ Pooled interval of 90% CIs across all models. Boldface values are significant (*p* < 0.05).
